# Evaluation of anaphylactic reactions involving carboplatin and paclitaxel reported to the FDA from 1970 to 2023

**DOI:** 10.1016/j.gore.2026.102122

**Published:** 2026-05-27

**Authors:** Halle Petrie, Taryn Boucher, Marta A. Crispens, Elizabeth J. Phillips, Cosby A. Stone

**Affiliations:** a Vanderbilt University School of Medicine, Nashville, TN, United States; b Vanderbilt Department of Obstetrics and Gynecology, Nashville, TN, United States; c University of Arizona Department of Obstetrics and Gynecology, Tucson, AZ, United States; d Vanderbilt Department of Infectious Diseases, Nashville, TN, United States; e Vanderbilt Department of Allergy, Pulmonary Critical Care Medicine, Nashville, TN, United States

**Keywords:** Chemotherapy, Anaphylaxis, Carboplatin, Paclitaxel, Endometrial cancer, Ovarian cancer

## Abstract

**Introduction:**

Anaphylaxis to carboplatin and paclitaxel are important and potentially life-threatening events that can disrupt chemotherapeutic treatment of gynecologic cancers. Using the FDA Adverse Event Report System (FAERS), we sought to establish the relative incidence of reports of anaphylactic reactions to these drugs compared to other drugs and identify potential associations with age and sex.

**Methods:**

Using the search terms “anaphylactic reaction”, “anaphylactic shock”, “anaphylactoid reaction”, or “anaphylactoid shock”, we searched FAERS for reports associated with carboplatin or paclitaxel from 1970 until October 2023. Fatal and non-fatal reactions were included. Linear regression evaluated trends in reported cases.

**Results:**

Of 84,702 anaphylactic events reported to the FDA during the study period, 4,988 (5.9%) were fatal. Carboplatin (1,245 reports) and paclitaxel (1,430 reports) ranked 14th and 9th among all drugs associated with anaphylaxis, and 10th and 3rd among those associated with fatal anaphylaxis (8.0% and 10.6% fatality, respectively). Most reactions occurred in adults aged 18–85 (carboplatin 79.0%; paclitaxel 83.7%), and the majority of affected patients were women (carboplatin 70.4%; paclitaxel 68.8%). From 2013 to 2022, yearly anaphylactic reaction means were 77.9 total and 6.6 fatal carboplatin reports (fatal ratio 0.08), and 84.4 total and 8.2 fatal paclitaxel reports (fatal ratio 0.10).

**Conclusion:**

The ratio of fatal to total anaphylactic events remained steady over the last decade, suggesting a fatality rate of 8–10% among reports. Additional research is needed to identify biomarkers and improve management of these reactions in gynecologic oncology patients.

## Highlights

• Paclitaxel ranked 3rd and carboplatin 10th in FDA fatal anaphylaxis reports.
• Paclitaxel ranked 9th and carboplatin 14th in all FDA anaphylaxis reports.
• Fatal anaphylaxis rate remained stable at 8–10% over the past decade.
• 68–70% of anaphylactic reactions to these agents occurred in women.
• Carboplatin and paclitaxel anaphylaxis reports increased significantly over time.

## 1 Background

Anaphylaxis to carboplatin and paclitaxel is an important and potentially life-threatening event that can disrupt the necessary chemotherapeutic treatment of gynecologic cancers. When the patient survives the anaphylactic reaction, treatment can subsequently be managed in two major ways: avoidance of the offending agent versus desensitization to allow continuation of treatment with the offending agent. (Bastin et al., 2026).

In a previous report using a restricted timeframe from 1999 to 2019, we observed that these two drugs were among the top reported drugs associated with both anaphylaxis and fatal anaphylaxis. (Yu et al., 2021) In recognition of the clinical relevance of these events, the Society of Gynecologic Oncology Education Committee recently developed a practice statement on the management of chemotherapy hypersensitivity reactions and desensitization regimens, reflecting a growing need for gynecologic oncology providers to recognize and manage these reactions in clinical practice. (Hall et al., 2023) Guidance from the American Academy of Allergy, Asthma, and Immunology and the American College of Allergy, Asthma and Immunology is also available to help clinicians to perform desensitization protocols. (Banerji et al., 2023, Khan et al., 2022, Broyles et al., 2020).

Immediate hypersensitivity reactions to chemotherapy fall into two mechanistic categories. IgE-mediated reactions involve sensitization to the active drug itself and typically occur after cumulative exposure. This is typical of carboplatin, which is formulated as a simple aqueous solution without additional excipients. (Tsao et al., 2022) Non-IgE-mediated infusion reactions are often complement-mediated and attributable to inactive solubilizing excipients, characteristically occurring on first or second exposure. In the case of paclitaxel, its solubilizing vehicle Cremophor EL (polyoxyl 35 castor oil) is the primary driver of hypersensitivity rather than the active drug itself. (Khan et al., 2022, Gelderblom et al., 2001) This mechanistic hypersensitivity distinction has shaped both formulation and premedication practice over time. Paclitaxel hypersensitivity rates of 25–30% in early trials fell to 2–4% after the introduction of a three-drug premedication regimen in the early 1990 s — dexamethasone, diphenhydramine, and an H2 blocker; this PO regimen was then simplified to single intravenous doses administered 30 min prior to infusion. (Khan et al., 2022, Bookman et al., 1997, Rowinsky and Donehower, 1995) The 2005 approval of nab-paclitaxel, a Cremophor-free formulation, further reduced infusion reaction. (Stinchcombe, 2007) Because carboplatin causes unpredictable IgE-mediated reactions, premedication has not been similarly standardized and is generally initiated only after an index reaction, often in conjunction with desensitization protocols. (Network and Clinical Practice Guidelines, 2025).

Given the importance of these drugs in treating gynecologic malignancies, we sought to establish the relative importance of these drugs amongst other key drugs associated with anaphylaxis across the entire timeframe of the publicly available FDA Adverse Event Report System (FAERS), and to uncover any potential associations of anaphylaxis reports with the patient factors of age and sex.

## 2 Methods

This study used publicly available, de-identified data from the FDA Adverse Event Reporting System (FAERS) and did not involve human subjects as defined by 45 CFR 46. It was therefore not subject to Institutional Review Board review.

Using the search terms “anaphylactic reaction”, “anaphylactic shock”, “anaphylactoid reaction”, or “anaphylactoid shock”, we searched the FDA’s FAERS database for reports associated with carboplatin or paclitaxel from 1970 until October 1st, 2023, during which time 27,096,432 total adverse events were reported. Both fatal and non-fatal reactions were included. We further specified timepoints to 2012 – 2023 for some of our analyses, as 2012 was the year that carboplatin/paclitaxel became the standard treatment for endometrial cancer, based on a randomized control trial by the Gynecologic Oncology Group. (Miller et al., 2020) Linear regression was applied to evaluate trends in reported cases.

## 3 Results

Of 27,096,432 adverse events reported during the study period, 84,702 (0.3%) were anaphylaxis reports, of which 4,988 (5.9%) were associated with fatality. Carboplatin was associated with 81,328 adverse events reported to the FDA; of those, 1,245 (1.5%) anaphylaxis events were reported, of which 100 (8.0%) were fatal. During the same timeframe, paclitaxel was associated with 89,240 adverse events reported to the FDA; of those, 1,430 (1.6%) anaphylaxis events were reported, of which 152 (10.6%) were fatal. When looking at all reports of anaphylaxis among all drugs, carboplatin and paclitaxel were the 14th and 9th most common drugs reported to the FDA in association with a report of anaphylaxis, respectively. When restricted to the 4,988 reports of anaphylaxis to the FDA in which the outcome was fatal, deaths associated with carboplatin and paclitaxel were the 10th and 3rd most common drugs reported in association with a fatal anaphylactic reaction, respectively.

***Reports of carboplatin anaphylaxis***.

Among 1,245 reports of anaphylaxis to carboplatin reported to the FDA, 877 (70.4%) were reported in women, 241 (19.4%) were reported in men, and 127 (10.2%) were reported without specified sex. There were 8 (0.6%) reported reactions in ages 2 months − 2 years, 15 (1.2%) in 3–––11 years, 10 (0.8%) in 12–––17 years, 584 (46.9%) in those 18–––64 years old, 400 (32.1%) in those 65–––85 years, and 13 (1.0%) in those older than 85, with 215 (17.3%) reports without specified age. 1078 (86.6%) anaphylaxis reports were made by healthcare providers, 139 (11.2%) by consumers, and 28 (2.3%) without a specified reporter.

**Fig. 1:**
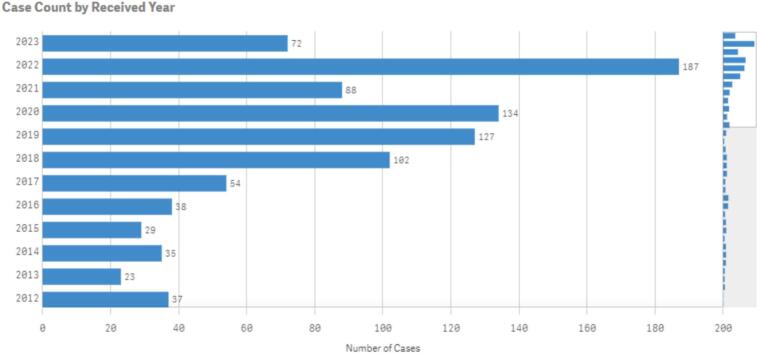
Reports of Anaphylaxis Associated with Carboplatin by Year. Note that reporting for 2023 represents an incomplete timeframe, only up to 10/1/23.

During the ten-year timeframe from 2013 − 2022, a mean of 77.9 anaphylactic reactions associated with carboplatin were reported to the FDA per year. **See** Fig. 1 **below**. When linear regression was applied, it suggested an upward linear trend in cases being reported, with a Beta-coefficient of 16 additional cases being reported per year (p < 0.005).

***Reports of fatal carboplatin anaphylaxis***.

Among 100 fatal anaphylactic reactions reported in association with carboplatin, 62 (62%) were reported in women, 23 (23%) were reported in men, and 15 (15%) were reported without specified sex. There were 2 (2%) fatal reactions in patients aged 3–––11 years old, 1 (1%) in 12–––17 years old, 44 (44%) in patients aged 18–––64, and 31 (31%) in patients aged 65–––85, with 22 (22%) reports without a specified age. The vast majority of cases were reported by healthcare providers (81%), with some reports by consumers (16%) and unspecified reporters (3%).

**Fig. 2:**
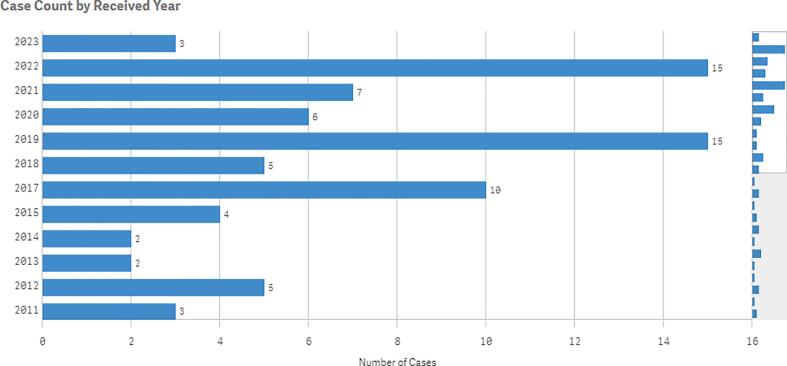
Reports of Fatal Anaphylaxis Associated with Carboplatin by Year. Note that reporting for 2023 represents an incomplete timeframe, only up to 10/1/23.

**Fig. 3:**
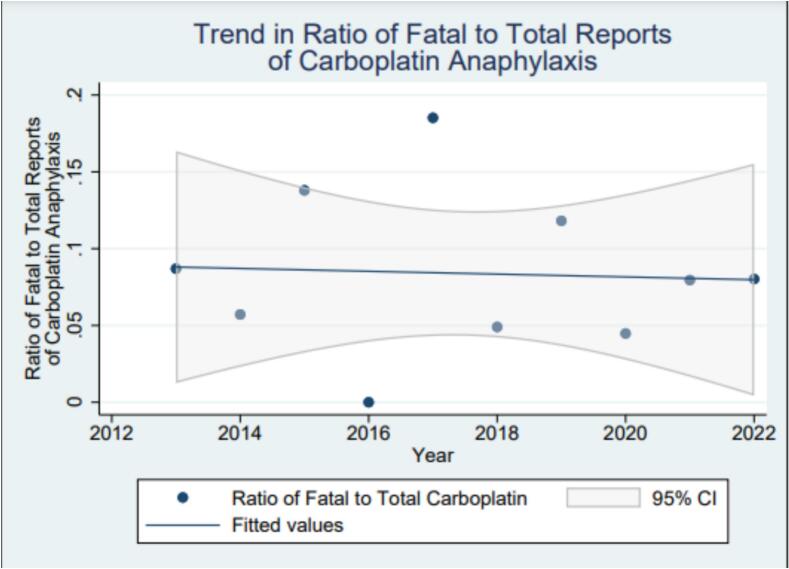
Trend in Ratio of Fatal to Total Reports of Carboplatin Anaphylaxis.

During the ten-year timeframe from 2013 − 2022, a mean of 6.6 fatal anaphylactic reactions associated with carboplatin were reported to the FDA per year. **See** Fig. 2 **below**. When linear regression was applied, it suggested an upward trend in reports of fatal cases, with a Beta coefficient of 1.2 additional cases per year (p = 0.02). At the same time, we observed that the annual ratio of 0.08 fatal anaphylaxis associated with carboplatin reported for every anaphylaxis report was flat, with a nonsignificant trend (p = 0.89). **See** Fig. 3 **below**.

***Reports of paclitaxel anaphylaxis***.

Among 1,430 reports of anaphylaxis to paclitaxel, 984 (68.8%) were reported in women, 336 (23.5%) were reported in men, and 110 (7.7%) were reported without specified sex. There were 2 (0.1%) reported reactions in ages 2 months − 2 years, 0 (0%) in 3–––11 years, 2 (0.1%) in 12–––17 years, 770 (53.8%) in those 18–64, 428 (29.9%) in those 65–85, and 1 (0.1%) in those older than 85, with 227 reports without specified age. 1,236 (86.4%) reports were made by healthcare professionals, with 154 (10.8%) reported by consumers and 40 (2.8%) without a specified reporter.

**Fig. 4:**
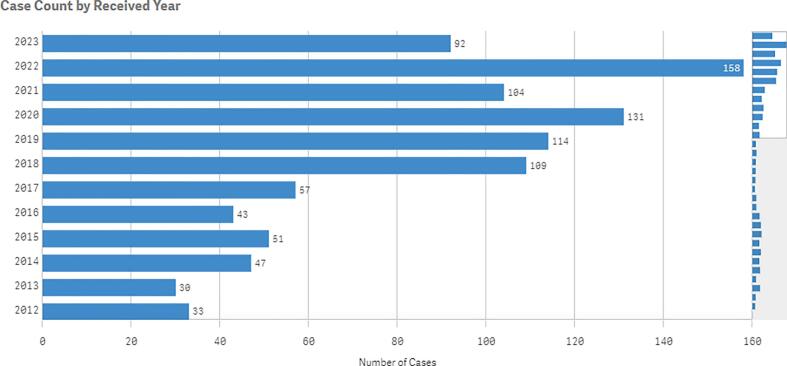
Reports of Anaphylaxis Associated with Paclitaxel by Year. Note that reporting for 2023 represents an incomplete timeframe, only up to 10/1/23.

During the ten-year timeframe from 2013 − 2022 a mean of 84.4 anaphylactic reactions associated with paclitaxel were reported to the FDA per year. **See** Fig. 4 **below**. When linear regression was applied, it suggested an upward linear trend in cases being reported, with a Beta-coefficient of 13.4 additional cases being reported per year (p < 0.005).

***Reports of fatal paclitaxel anaphylaxis***.

Among 152 fatal anaphylactic reactions reported in association with paclitaxel, 96 (63.2%) were reported in women, 48 (31.6%) were reported in men, and 8 (5.3%) were reported without specified sex. There were 0 (0%) fatal reactions reported in patients 0–––17 years of age, 75 (49.3%) in those aged 18–––64, 61 (40.1%) in those aged 65–––85, and 16 (10.5%) reports in which age was not specified. 125 (82.2%) reports were initiated by healthcare providers, 23 (15.1%) by consumers, and 4 (2.6%) did not specify their source.

**Fig. 5:**
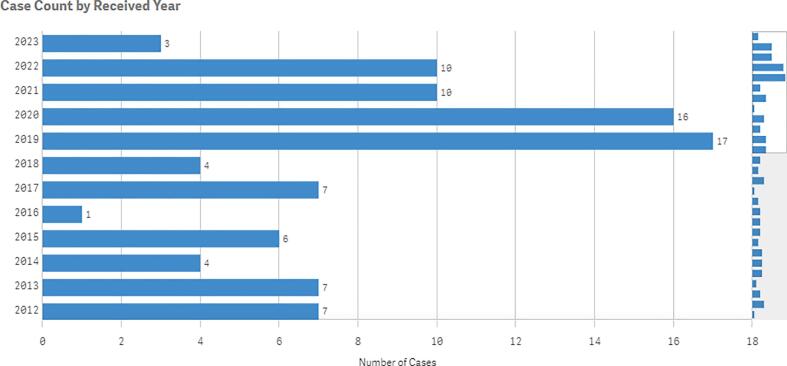
Reports of Fatal Anaphylaxis Associated with Paclitaxel by Year. Note that reporting for 2023 represents an incomplete timeframe, only up to 10/1/23.

**Fig. 6:**
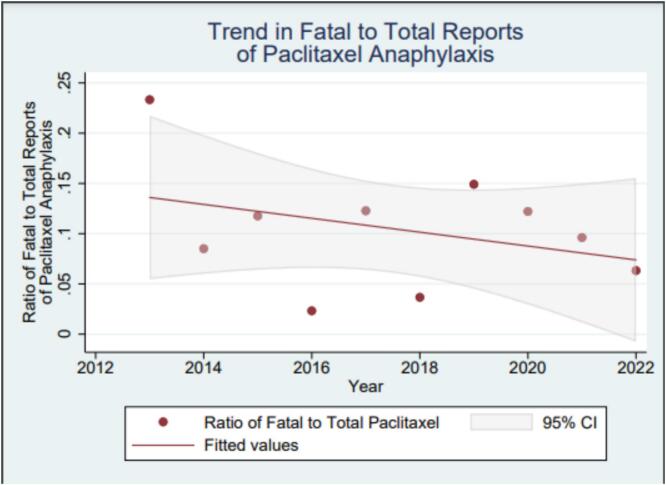
Trend in Ratio of Fatal to Total Reports of Paclitaxel Anaphylaxis.

During the ten-year timeframe from 2013 − 2022 a mean of 8.2 fatal anaphylactic reactions associated with paclitaxel were reported to the FDA per year. **See** Fig. 5 **below**. When linear regression was applied, it suggested a nonsignificant upward trend in reports of fatal cases, with a Beta-coefficient of 0.99 additional cases per year (p = 0.08). At the same time, we observed an average annual ratio of 0.10 fatal anaphylaxis associated with paclitaxel reported for every anaphylaxis report, with a downward linear trend that was not statistically significant (p = 0.33). **See** Fig. 6 **below**.

## 4 Discussion

Across the full timeframe of FDA Adverse Event Report System data from 1970 until 2023, carboplatin and paclitaxel were the 14th and 9th most common drugs reported to the FDA in association with anaphylaxis, and they were the 10th and 3rd most common drugs reported to the FDA in association with fatal anaphylaxis. Reports of both anaphylaxis and fatal anaphylaxis were more common in adults. Furthermore, 68–70% of anaphylactic reactions occurred in women. This disproportionate impact on adult women may reflect the use of these agents in cancers that preferentially affect women, including gynecologic malignancies, breast cancer, and lung cancer, in which women are increasingly represented. We observed upward trends in reports of anaphylaxis and fatal anaphylaxis to both carboplatin and paclitaxel over time. This may reflect increased drug administration, greater reporting of anaphylaxis to the FDA, or both. Recent data have shown rising rates of endometrial cancer in patients under 50 and increased prescribing of systemic chemotherapy for endometrial cancer between 2005–2008 and 2017–2020, supporting a growing population exposed to these agents. (Adekanmbi et al., 2024) Despite rising absolute numbers, the ratio of fatal to total anaphylaxis reports remained stable, suggesting that fatal anaphylaxis occurs in approximately 8–10% of those who react.

Unlike many drugs listed in the database, most reports of anaphylaxis to carboplatin and paclitaxel were made by healthcare providers, likely because these reactions are observed in monitored infusion centers. This clinical setting may facilitate rapid recognition and intervention, suggesting that the true severity of these reactions may be underestimated by fatality rates alone. (Sato-DiLorenzo et al., 2022).

At the same time, the greater context is that carboplatin and paclitaxel are indispensable in the treatment of gynecologic malignancies due to lack of superior alternatives. Hypersensitivity reactions threaten discontinuation of these life-sustaining therapies. In ovarian cancer, the carboplatin/paclitaxel regimen is the standard first-line chemotherapy for most epithelial ovarian, fallopian tube, and peritoneal cancers across all stages, including high-grade serous carcinoma, grade II-III endometrioid carcinoma, clear cell carcinoma, and carcinosarcoma. In the setting of ovarian/fallopian tube/peritoneal cancer recurrence with platinum sensitivity, carboplatin/paclitaxel with the potential addition of bevacizumab is recommended. Beyond its combination with paclitaxel, carboplatin is used alongside other chemotherapy agents – such as pegylated doxorubicin and gemcitabine – across multiple lines of treatment, increasing cumulative exposure and thus the risk of hypersensitivity. (Network and Clinical Practice Guidelines, 2025) Hypersensitivity can effectively render platinum-sensitive patients platinum-ineligible, compromising outcomes; switching to non-platinum agents in platinum-sensitive disease has been associated with significantly lower response rates and worse progression-free survival. (Caruso et al., 2025).

While drug substitution post-allergic reaction is sometimes attempted, the evidence varies by drug class. For platinum agents, cross-reactivity between carboplatin and cisplatin is low, and cisplatin has been safely administered in the majority of carboplatin-allergic patients; however, cisplatin's more severe nonhematologic toxicity profile – including nephrotoxicity, neurotoxicity, and ototoxicity – makes it an imperfect substitute. (Bookman et al., 1997, Stinchcombe, 2007, Network and Clinical Practice Guidelines, 2025) Paclitaxel can be substituted with nab-paclitaxel or docetaxel. In taxanes, hypersensitivity reactions are more often attributed to inactive ingredients rather than the active drug itself. Thus, nab-paclitaxel has been safely used in most patients with prior paclitaxel reactions but is not universally safe. Cross-reactivity between paclitaxel and docetaxel ranges from 50 to 90% in published studies and can be life-threatening. (Khan et al., 2022, Bookman et al., 1997) Despite these options, desensitization to the original agent remains the preferred approach when the implicated drug is first-line therapy.

Carboplatin and paclitaxel also serve as the first-line standard for high-risk early stage, advanced, and recurrent endometrial carcinoma. In advanced (stage III and IV) and recurrent disease, these agents form the chemotherapy backbone for various immunotherapy combinations. In high-risk early-stage disease – such as in patients with aggressive histology – this combination is used as adjuvant treatment. (Network and Clinical Practice Guidelines, 2025, Miller et al., 2020) In cervical cancer, these agents are used for recurrent and/or metastatic disease as well as induction before chemoradiation for locally advanced disease. (Network and Clinical Practice Guidelines, 2025).

The timing of carboplatin hypersensitivity makes it especially relevant in the treatment of gynecologic malignancies. While paclitaxel hypersensitivity reactions most commonly occur at initial exposure, carboplatin reactions typically develop after repeated exposures. (Rowinsky and Donehower, 1995, Tai et al., 2017) Because chemotherapy is administered in cycles, many patients face a cumulative and increasing risk of carboplatin reactions over time.

A major limitation of FAERS data is its voluntary reporting and the lack of denominator data. There is a high risk of selection and reporting bias that favors more serious observed events, so these data should not be used to estimate the true incidence of anaphylaxis or fatal anaphylaxis associated with these drugs. Conversely, it is also likely that many of the severe events that occur in current practice are not reported. Drugs that are closely monitored during their provision – in intraoperative settings and infusion centers – could also have a lower rate of fatal anaphylaxis due to earlier recognition and treatment of severe reactions. More broadly, relying on patient, physician, pharmaceutical company, or literature-initiated reports to the FDA does not provide an exact estimate of the true incidence of anaphylaxis to a drug; these data are inherently limited by under-reporting of cases, indeterminate causality, and the potential existence of duplicate reports. Additionally, although the FAERS database extends to 1970, carboplatin and paclitaxel were not FDA-approved until 1989 and 1992, respectively; pre-approval reports were negligible and did not alter our findings.

FAERS also does not systematically capture manufacturer, lot number, or formulation details, preventing batch-level analysis of whether specific manufacturing changes contributed to variation in reports across the years. Notably, paclitaxel anaphylaxis reports continued to rise after the 2005 FDA approval of nab-paclitaxel, suggesting that solvent-based paclitaxel has remained in widespread use despite the availability of a Cremophor-free alternative. Furthermore, because each FAERS report represents a discrete event rather than a longitudinal patient record, we are unable to determine downstream clinical management, including whether patients underwent rechallenge or formal desensitization.

Furthermore, FAERS lacks ethnicity and geographical data that would allow for studies of regional, socioeconomic, and urban versus rural trends, which are important considerations when evaluating all adverse drug reactions alongside their ecological and genetic contributors. Differences in ethnicity and geography contribute to disparities in access to desensitization protocols and allergy/immunology expertise, which varies considerably across centers. (Hung et al., 2022) As a result, patients treated at smaller or lower-resourced centers may be more likely to permanently discontinue first-line chemotherapy after an allergic reaction, potentially impacting morbidity and mortality. (Bastin et al., 2026) This is particularly concerning in ovarian cancer, where carboplatin and paclitaxel are used in combination across multiple stages of treatment – primary, neoadjuvant, adjuvant, recurrence – and discontinuation of these agents can significantly impact survival. (Caruso et al., 2025, Gaillard et al., 2025) Despite these limitations, FAERS data serve as an important source of information about what is being reported in healthcare settings across the US and the world.

Desensitization strategies currently exist to mitigate the harms of allergy to both carboplatin and paclitaxel, even in cases of anaphylaxis, and have been shown to be 95–100% effective in allowing future cycle continuation. (Bastin et al., 2026, Zwimpfer et al., 2024, Liang et al., 2025, Bulut and Yegin, 2023, Sağun et al., 2026) In fact, in a study of 241 patients with gynecologic cancer with hypersensitivity to either carboplatin or paclitaxel, desensitization facilitated continuation of the allergy-causing agent in all patients. (Zwimpfer et al., 2024) Moreover, desensitization to carboplatin has been associated with longer progression-free survival compared to replacement with cisplatin, highlighting the clinical benefit of maintaining the first-line regimen. (Bastin et al., 2026) Unfortunately, these strategies are not universally available due to the lack of expertise in the relevant protocols and modified management. The SGO Education Committee's recent practice statement on chemotherapy hypersensitivity management represents one effort to address this gap. (Hall et al., 2023).

Lastly, there is currently no testing strategy that can easily diagnose the potential for an allergic reaction to carboplatin or paclitaxel before it occurs. Additional research is crucial for establishing biomarkers of these allergies prior to severe reaction and to improve our existing management, particularly in gynecologic oncology, where these agents are central to treatment.

## 5 Conclusions

In a focused review of publicly available FAERS data, we evaluated reports of anaphylaxis to carboplatin and paclitaxel, two chemotherapeutic drugs essential in the treatment of gynecologic malignancies. We found that these drugs are among the leading causes of fatal anaphylaxis reported to the FDA and that such events are more likely to happen to adult women. Given the 8–10% rate of reported fatality amongst these reports, additional attention and research on biomarkers, desensitization, and other forms of modified allergy management are warranted.

## CRediT authorship contribution statement

**Halle Petrie:** Writing – review & editing, Writing – original draft, Validation, Conceptualization. **Taryn Boucher:** Writing – review & editing, Writing – original draft, Visualization, Methodology, Formal analysis, Data curation, Conceptualization. **Marta A. Crispens:** Writing – review & editing. **Elizabeth J. Phillips:** Writing – review & editing. **Cosby A. Stone:** .

## Declaration of Competing Interest

The authors declare that they have no known competing financial interests or personal relationships that could have appeared to influence the work reported in this paper.
